# Gram-scale green synthesis of a highly stable cationic covalent organic framework for efficient and selective removal of ReO_4_^−^/^99^TcO_4_^−^†

**DOI:** 10.1039/d4ta06442a

**Published:** 2024-11-05

**Authors:** Changxia Li, Justyna Florek, Patrick Guggenberger, Freddy Kleitz

**Affiliations:** a School of Chemistry and Molecular Engineering, Nanjing Tech University 211816 Nanjing China; b Department of Functional Materials and Catalysis, Faculty of Chemistry, University of Vienna 1090 Vienna Austria changxia.li@univie.ac.at freddy.kleitz@univie.ac.at; c Vienna Doctoral School in Chemistry (DoSChem), University of Vienna 1090 Vienna Austria

## Abstract

Covalent organic frameworks (COFs) have developed as efficient and selective adsorbents to mitigate ^99^TcO_4_^−^ contamination. However, the eco-friendly and scalable production of COF-based adsorbents for the removal of ^99^TcO_4_^−^ has not yet been reported. This study explores the potential of a cationic COF (TpDB-COF) synthesized *via* a green hydrothermal method, achieving gram-scale yields per batch, thereby addressing a significant limitation of existing COF production methods. The TpDB-COF demonstrates an exceptional stability in strongly acidic conditions (2 weeks in 3 M HNO_3_), as well as in various organic solvents, making it suitable for harsh nuclear waste environments. Adsorption experiments using ReO_4_^−^ as a surrogate for ^99^TcO_4_^−^ show rapid adsorption kinetics, reaching nearly 100% removal efficiency within 1 min (with initial concentration of 28 ppm at a solid-to-liquid ratio of 1 g L^−1^), a maximum adsorption capacity of 570 mg g^−1^ and excellent stability. Moreover, the COF maintains high selectivity for ReO_4_^−^ even in the presence of competing anions such as SO_4_^2−^ and NO_3_^−^. These findings highlight that the hydrothermal synthesis is an effective method to synthesize COF adsorbents for efficient removal of ^99^TcO_4_^−^ and offers a sustainable approach for practical applications.

## Abstract 2

Green hydrothermal synthesis achieves gram-scale production of a cationic COF sorbent with high stability for the rapid and selective removal of ^99^TcO_4_^−^ contaminants.

## Graphical abstract

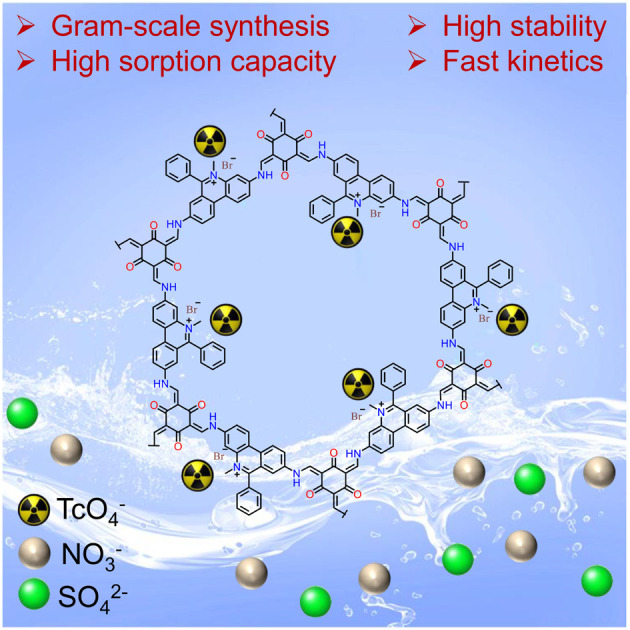

## Introduction

Technetium-99 (Tc-99) is a significant radioactive contaminant resulting from the nuclear fission of uranium-235 and plutonium-239 in nuclear reactors and nuclear weapons.^1^ As a fission product, Tc-99 poses substantial environmental and health threats due to its long half-life of approximately 2.13 × 10^5^ years and its high mobility in the environment.^2,3^ The pertechnetate anion (^99^TcO_4_^−^), a common chemical form of technetium in aqueous environments, is highly soluble (11.3 M at 20 °C) and chemically stable, complicating its capture from contaminated sites and waste streams.^2,4^ Therefore, developing efficient and selective materials for ^99^TcO_4_^−^ removal is of paramount importance for ensuring environmental safety and effective nuclear waste management.

To address this challenging issue, several types of cationic solid adsorbents including anion-exchange resins,^5,6^ inorganic cationic materials,^7–10^ metal–organic frameworks (MOFs)^11–15^ and covalent organic frameworks (COFs)^16–24^ have been investigated for their potential in ^99^TcO_4_^−^ capture *via* the ion-exchange method. Considering the radioactive nature of ^99^TcO_4_^−^, ReO_4_^−^ is usually used as a surrogate in laboratory studies due to their similar chemical properties. Among the reported adsorbents, inorganic materials and anion-exchange resins usually suffer from low uptake capacity and poor selectivity due to the limitation of their irregular porosity. On the other hand, MOFs feature highly tunable porosity. However, their poor chemical stability under strongly acidic conditions (*e.g.*, 3 M HNO_3_, which is required in spent fuel reprocessing) seriously impedes their practical applications.^13–15^

Covalent organic frameworks (COFs) have emerged as an important family of porous materials due to their high specific surface area, tunable porosity, and structural versatility.^25–30^ Compared to most MOFs, COFs are formed by the covalent bonding of organic molecules and therefore exhibit enhanced stability, especially the β-ketoenamine-linked COFs, which can maintain stability under extreme conditions including strong acidity and alkalinity.^31^ Generally, the scaffold charge affects the uptake of cationic and anionic species from solution. Positively charged scaffolds can enhance anion adsorption through electrostatic attraction, and the chemical environment within the scaffolds also influences selective uptake based on charge.^32,33^ Recent research has demonstrated that cationic COFs can be highly effective for the selective adsorption of ^99^TcO_4_^−^/ReO_4_^−^.^18,34^ The positive charge on the COFs enhances the electrostatic interaction with the negatively charged ^99^TcO_4_^−^/ReO_4_^−^ ions, improving the adsorption efficiency and selectivity. Various types of cationic COFs have been developed, each with unique structural and functional properties. For example, Wang's group reported a two-dimensional (2D) conjugated cationic COF, which possessed good stability in acids and high uptake capacity of 702.4 mg g^−1^ for ReO_4_^−^.^20^ Another example is ionic three-dimensional (3D) sp^2^ carbon-linked COFs constructed by Qiu's group.^21^ These COFs exhibited a high adsorption capacity (542.3 mg g^−1^ for ReO_4_^−^) and rapid adsorption kinetics, achieving a quantitative ReO_4_^−^ removal within 30 s. Despite the promising adsorption capacities, current COF-based adsorbents are typically synthesized using solvothermal methods with complicated synthesis procedures and non-environmentally friendly reagents. These approaches often result in limited production scales, with each batch yielding only around 100 mg (Table S1†), severely hindering their practical applications.

In our study, we address this limitation of the current cationic COF synthesis methods by employing a green hydrothermal synthesis route. This method is simple, efficient, and green, achieving gram-scale production of COF per batch. The synthesized COF exhibits excellent acid–base stability, particularly in highly acidic solutions (stable for 2 weeks in 3 M HNO_3_ solution), making it suitable for long-term applications in harsh environments typical of nuclear waste repositories. Notably, the cationic COF exhibited rapid sorption kinetics, high uptake capacity, good selectivity, and recyclability toward ReO_4_^−^ removal. The green synthesis approach and the COF's stability and performance in acidic conditions make it a viable option for practical applications in mitigating ^99^TcO_4_^−^ contamination.

## Experimental section

### Synthesis of TpDB-COF

1,3,5-triformylphloroglucinol (Tp) was synthesized according to the reported method.^35^ TpDB-COF was synthesized following our previously developed hydrothermal method^35–38^ with some modifications. 2.5 g of *p*-toluene sulfonic acid (PTSA, Sigma-Aldrich) and 856 mg of dimidium bromide (DB, TCI) were mixed, then 5 mL of water was added drop by drop. The mixture was ground thoroughly using a mortar, then transferred into a bottle, and 15 mL of water was added. The mixture was shaken well in a vortex shaker for 5 min. Then, 315 mg of Tp was added and shaken for another 20 min. The red mixture was transferred into the autoclave with another 5 mL water and kept in the oven at 125 °C for 24 h. Then, the red precipitate was filtered and sequentially washed with 3 M HNO_3_ and water. Finally, the collected solid was Soxhlet extracted with THF and dried at 100 °C to get TpDB-COF in ∼96% yield.

## Results and discussion

**Fig. 1:**
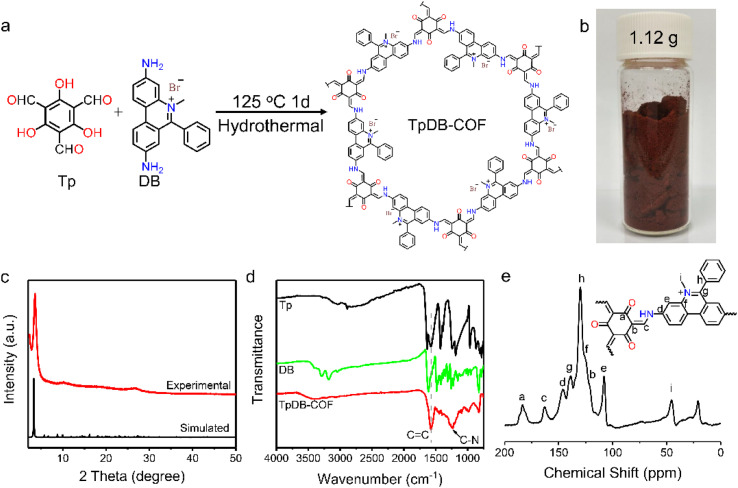
(a) Synthesis of TpDB-COF. (b) Photograph of one-batch synthesis of TpDB-COF. (c) Experimental and simulated powder XRD pattern of TpDB-COF. (d) FTIR spectra of Tp, DB, and TpDB-COF. (e) Solid-state ^13^C CP-MAS NMR spectrum of TpDB-COF.

The facile synthesis of TpDB-COF was realized from 1,3,5-triformylphloroglucinol (Tp) and dimidium bromide (DB) with PTSA as catalyst in a hydrothermal route at 125 °C for 1 day (Fig. 1a). The TpDB-COF was achieved as a dark red powder with a yield of 96% (∼1.12 g per batch, as shown in Fig. 1b), much higher than the solvothermal method (76% yield, ∼0.059 g per batch).^39^ The formation of TpDB-COF was identified by powder X-ray diffraction (PXRD), Fourier-transform infrared spectroscopy (FTIR), and ^13^C solid-state nuclear magnetic resonance (NMR) spectroscopy. The XRD pattern displays a strong diffraction peak at 3.56°, corresponding to the (100) plane, which agrees with the simulated AA stacking mode (Fig. 1c). The high signal-to-noise ratio of (100) peak indicates the high crystallinity of TpDB-COF. The FTIR spectra demonstrate the disappearance of the N–H stretching vibration band at 3100–3400 cm^−1^ and the aldehyde group stretching vibration at around 1638 cm^−1^, indicating the Schiff-based condensation reaction (Fig. 1d). The appearance of the stretching vibrations for the C=C (1576 cm^−1^) and C–N (1251 cm^−1^) bonds and the absence of the characteristic C=N bond around 2220 cm^−1^ reflect the existence of the keto form, which was further validated by ^13^C cross-polarization magic angle spinning (CP-MAS) NMR spectroscopy through the presence of the peak at ∼183 ppm (Fig. 1e).^40,41^

**Fig. 2:**
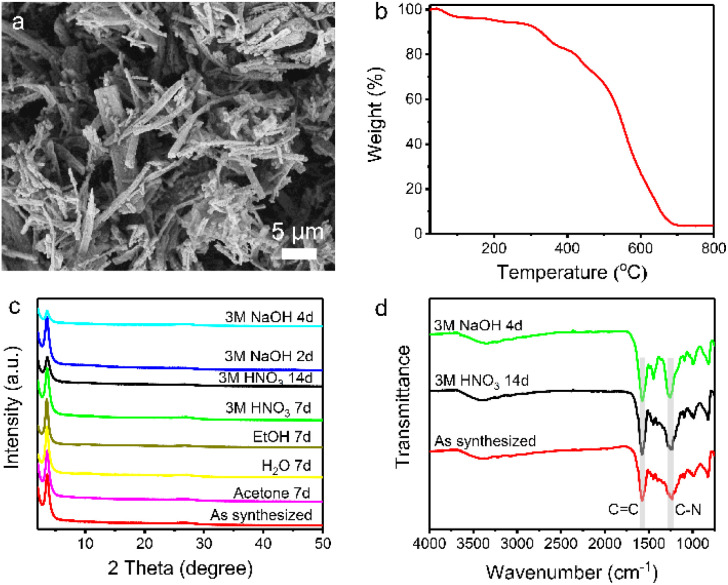
(a) SEM image and (b) TGA curve of TpDB-COF. (c) PXRD patterns of TpDB-COF after treatment in various solvents, acids, and bases. (d) FT-IR spectra of TpDB-COF after treatment with 3 M HNO_3_ and 3 M NaOH.

Scanning electron microscopy (SEM) reveals the rod-like morphology of TpDB-COF (Fig. 2a). The thermal stability of TpDB-COF was investigated by thermogravimetric analysis (TGA) under air flow. The mass loss below 100 °C corresponds to lattice water and the TpDB-COF remains stable up to 300 °C (Fig. 2b). The porosity was revealed by N_2_ physisorption measurement at −196 °C (77 K) (Fig. S1 and S2†). The calculated Brunauer–Emmett–Teller (BET) surface area is 68 m^2^ g^−1^. The average pore width is 1.2 nm as calculated by the quenched solid density functional theory (QSDFT). Compared to the previous solvothermal method (495 m^2^ g^−1^)^39^ and microwave irradiation solvothermal method (747 m^2^ g^−1^)^40^ using the same monomers, the BET surface area of TpDB-COF prepared using the hydrothermal method appears to be lower. CO_2_ adsorption measurements at room temperature (25 °C) showed a Langmuir surface area of 124 m^2^ g^−1^ (Fig. S3†), which is significantly higher than the BET surface area obtained from N_2_ physisorption. This discrepancy suggests that the COF framework may undergo partial pore closure under the conditions used for N_2_ adsorption at −196 °C, likely due to the flexibility of the structure and the potential for restricted pore accessibility at low temperatures. In contrast, CO_2_ adsorption at room temperatures enables probing of ultramicropores down to 0.35 nm and provides more realistic representation of accessible surface for ReO_4_^−^ ions (diameter 0.26 nm).^42,43^

The chemical stability was checked by dispersing the as-synthesized sample in different organic solvents and acid/base solutions. TpDB-COF possesses excellent chemical stability as demonstrated by the XRD patterns that remained almost unchanged compared to that of the pristine material after immersion into acid or organic solvents for 7 days (Fig. 2c). Even after 14 days in 3 M HNO_3_, the COF still retains its ordered crystalline structure. Furthermore, TpDB-COF also displays a certain stability in a strong basic solution. The XRD pattern matches well with that of the original one after 2 days but displays a decreased crystallinity after 4 days. TEM analysis reveals that the TpDB-COF nanorods exhibit a hollow structure (Fig. S4a†). After subjecting the material to different acid and base stability tests, no significant changes in morphology were observed (Fig. S4b and c†). Furthermore, FTIR also proved the exceptional chemical stability of TpDB-COF in highly acidic and basic solutions, with characteristic vibrational peaks similar to the as-synthesized sample (Fig. 2d). This excellent stability across a range of harsh conditions makes the prepared COF a promising candidate for practical applications of ^99^TcO_4_^−^ capture in environments where it may be exposed to various chemical conditions, such as those encountered in nuclear waste management and environmental remediation.

**Fig. 3:**
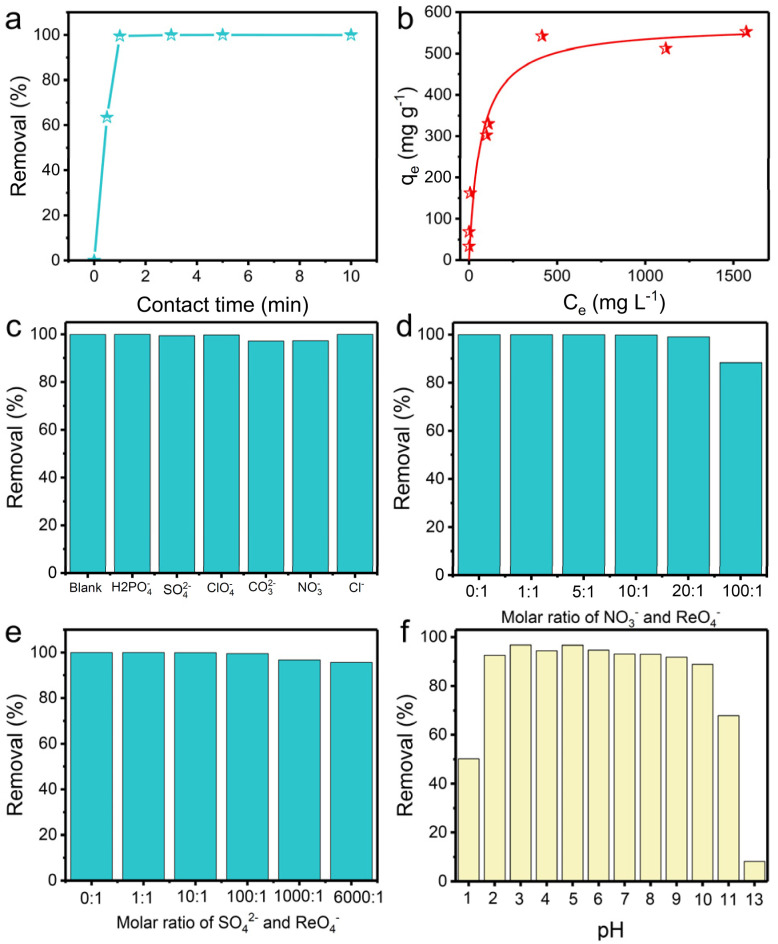
(a) Adsorption kinetics of ReO_4_^−^ by TpDB-COF. (b) Adsorption isotherm of TpDB-COF for ReO_4_^−^ uptake at room temperature. (c) Effect of competing anions on the removal of ReO_4_^−^ by TpDB-COF. Effect of excess (d) NO_3_^−^ and (e) SO_4_^2−^ on the ReO_4_^−^ exchange. (f) Effect of pH on the removal of ReO_4_^−^ by TpDB-COF (initial concentration of Re: ∼200 ppm; solid/liquid ratio of 1 mg mL^−1^).

Anion-exchange experiments were carried out to gain insight into the performance of TpDB-COF for the adsorption of ReO_4_^−^ as a surrogate for TcO_4_^−^. The adsorption kinetics were evaluated by soaking 10 mg of TpDB-COF in 10 mL of an aqueous solution containing 28 ppm ReO_4_^−^ at room temperature. Fig. 3a shows the removal percentage of ReO_4_^−^ as a function of contact time. The adsorption process of TpDB-COF is extremely rapid, reaching equilibrium within 1 min. This suggests that the COF material has a high affinity to ReO_4_^−^ and that the adsorption sites are readily accessible. The rapid kinetics are advantageous for practical applications because the short contact time could lower the nuclear leakage risk and subsequent environmental contamination. The morphology and structure of TpDB-COF show no obvious change after the sorption of ReO_4_^−^ (Fig. S5†). The elemental mapping visually confirms the ion-exchange behaviour of Br^−^ with ReO_4_^−^, and the resulting homogeneous distribution of ReO_4_^−^ in TpDB-COF (Fig. S6†).

The adsorption isotherm experiment at 25 °C was performed to evaluate the ReO_4_^−^ trapping capacity of TpDB-COF. As depicted in Fig. 3b, the relationship between the equilibrium concentration of ReO_4_^−^ (*C*_e_) and the adsorption capacity (*q*_e_) fits well with a Langmuir adsorption model and the maximum adsorption capacity is calculated to be 570 mg g^−1^. Compared with polymeric anion-exchange resins,^5,6,44,45^ inorganic materials^7–10,46–48^ and MOFs,^11–15,49,50^ TpDB-COF exhibits significantly higher capacity for ReO_4_^−^ uptake. This value is also comparable with other reported COF-based sorbents (Table S1†).^16–24,51,52^ However, the recently reported COF/MOF-based sorbents were mainly prepared on a milligram scale using toxic organic solvents as reagents, while TpDB-COF was produced on a gram-scale using only water as a reaction solvent (Table S1†).

In effluents contaminated with certain nuclides, various competing anions are present, such as H_2_PO_4_^−^, SO_4_^2−^, ClO_4_^−^, CO_3_^2−^, NO_3_^−^, Cl^−^, *etc.* These anions, especially SO_4_^2−^ and CO_3_^2−^, have stronger electrostatic interactions with the sorbents due to their higher charge density, which significantly affect the selective trapping of TcO_4_^−^. Therefore, it is important to determine the influence of these competing anions. As shown in Fig. 3c, the ReO_4_^−^ removal efficiency remained above 97%, indicating the high selectivity of the TpDB-COF for ReO_4_^−^ even in the presence of competing anions (with an equivalent stoichiometric ratio).

Selectivity for ReO_4_^−^ capture was further investigated in solutions of different equivalents of NO_3_^−^ and SO_4_^2−^. With increasing molar ratio of NO_3_^−^ to ReO_4_^−^ from 1 : 1 to 100 : 1, the removal efficiency of ReO_4_^−^ slightly decreases but remains above 88% (Fig. 3d), which is much higher than values reported for SCU-COF-1 (60%),^20^ polyILs@MOF@COF (74.4%),^49^ and imidazolium-based ionic liquid grafted COF (50%).^17^ The high selectivity suggests that while NO_3_^−^ ions compete for adsorption sites, the COF material still preferentially adsorbs ReO_4_^−^. In a similar way, the removal efficiency of ReO_4_^−^ is only marginally affected by the presence of SO_4_^2−^ at varying molar ratios up to 6000 : 1 (Fig. 3e). The removal efficiency remains high, above 95%, even at the highest concentration of SO_4_^2−^. This value is significantly higher than those of most reported adsorbents, such as SCU-103 (82%),^14^ imidazolium-based ionic liquid grafted COF (65%),^17^ and SCU-CPN-1 (64%),^53^ which further underscores the stoichiometric ratio for ReO_4_^−^ ions.

High extraction ability of ^99^TcO_4_^−^ in extreme conditions, such as various pH environments, is highly desirable. The capture capacity of TpDB-COF for ReO_4_^−^ at different pH levels was confirmed in 200 ppm of ReO_4_^−^ solutions at a solid/liquid ratio of 1 mg mL^−1^ (Fig. 3f). The removal percentages stayed high (>89%) across a broad pH range of 2–10. Even in 0.1 M HNO_3_ (pH 1), TpDB-COF achieves an ReO_4_^−^ uptake efficiency of 50%. However, the capture efficiency dropped to only 8% in 0.1 M NaOH aqueous solution (pH 13). These results preliminarily indicate that TpDB-COF exhibit better adsorption properties under acidic conditions.

**Fig. 4:**
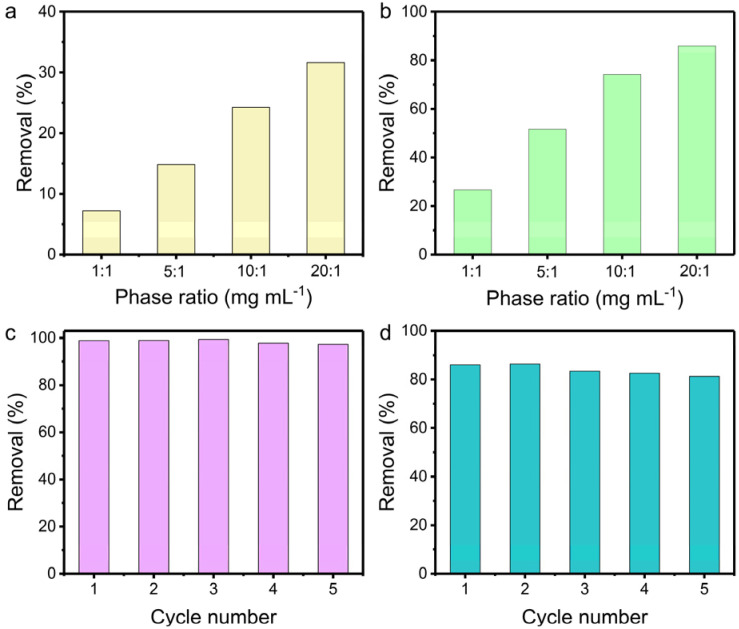
Removal of ReO_4_^−^ by TpDB-COF with various solid/liquid ratios in (a) 1 M NaOH and (b) 1 M HNO_3_ aqueous solution containing ∼200 ppm ReO_4_^−^. (c) Reusability of TpDB-COF in the removal of ReO_4_^−^ at pH 7 with an initial concentration of ReO_4_^−^ ∼28 ppm (solid/liquid ratio of 1 mg mL^−1^). (d) Reusability of TpDB-COF in the removal of ReO_4_^−^ in 1 M HNO_3_ with an initial concentration of ReO_4_^−^ ∼200 ppm (solid/liquid ratio of 20 mg mL^−1^).

The influence of the solid/liquid ratio on the uptake efficiency was probed in 1 M NaOH and 1 M HNO_3_ aqueous solution containing 200 ppm of ReO_4_^−^, respectively. The removal efficiency in 1 M NaOH increased from 7% to 32% with the solid/liquid ratio increasing from 1 to 20 mg mL^−1^ (Fig. 4a). Notably, TpDB-COF achieves higher ReO_4_^−^ uptake in 1 M HNO_3_ solution (Fig. 4b). As the solid/liquid ratio varies from 1 to 20, the corresponding capture percentages improve from 27% to 86%. This further demonstrates that TpDB-COF is indeed viable for ^99^TcO_4_^−^/ReO_4_^−^ separation from acidic nuclear waste.

Furthermore, the reusability of TpDB-COF was assessed in neutral solution containing 28 ppm of ReO_4_^−^. The ReO_4_^−^ ion-exchanged TpDB-COF can be effectively eluted by 1 M NaBr solutions. This elution not only displaces the ReO_4_^−^ ions through an ion-exchange process but also restores the COF to its original state, with Br^−^ as the counter-ion. After five sorption/desorption cycles, the removal efficiency of TpDB-COF almost remained unaltered (Fig. 4c). More impressively, TpDB-COF also showed excellent regeneration performance after ReO_4_^−^ removal in 1 M HNO_3_ at a solid/liquid ratio of 20 mg mL^−1^. Even after five sorption/desorption cycles, the removal rate still exceeded 81% (Fig. 4d). FTIR and PXRD analyses revealed that the TpDB-COF sorbent after elution of the analytes could revert to its original form (Fig. S7 and S8†). The high removal efficiency and stability in highly acidic conditions further demonstrate that TpDB-COF is particularly suitable for applications involving acidic nuclear waste streams.

## Conclusions

This work establishes a green, simple, and scalable approach to synthesize a cationic COF, which was produced in high yield at a gram-scale. The synthesized TpDB-COF can be applied for the adsorption of ^99^TcO_4_^−^/ReO_4_^−^ ions, due to the rapid adsorption kinetics, high adsorption capacity, and remarkable selectivity even in the presence of competing anions. Its stability in strongly acidic conditions further enhances its suitability for practical applications in nuclear waste management and ensures the durability and reliability of the adsorbent over prolonged periods. These results indicate that the TpDB-COF is a promising candidate for the effective removal of ^99^TcO_4_^−^ from contaminated environments, providing a viable solution to mitigate the environmental impact of technetium. Furthermore, it is expected that such highly robust and scalable COF material will be able to overcome other challenges in radionuclide separation during nuclear waste disposal, such as lanthanide/actinide separation.

## Data availability

The authors confirm that the data supporting this article have been included as part of the ESI.†

## Author contributions

Changxia Li: conceptualization, data curation, investigation, methodology, funding acquisition, project administration, writing – original draft, writing – review & editing. Justyna Florek: investigation, writing – review & editing. Patrick Guggenberger: investigation, writing – review & editing. Freddy Kleitz: conceptualization, funding acquisition, project administration, supervision, writing – original draft, writing – review & editing.

## Conflicts of interest

There are no conflicts to declare.

## Supplementary Material

TA-013-D4TA06442A-s001
