# Supplementary material for: Gram-scale green synthesis of a highly stable cationic covalent organic framework for efficient and selective removal of ReO4−/99TcO4−
Source: J Mater Chem A Mater. 2024 Nov 5;13(1):214–9. doi: 10.1039/d4ta06442a (PMC11566665; doi:10.1039/d4ta06442a)
Supplement: TA-013-D4TA06442A-s001 [file TA-013-D4TA06442A-s001.pdf]

Supplementary Information (SI) for Journal of Materials Chemistry A.
This journal is © The Royal Society of Chemistry 2024

Supplementary Information

Gram-scale green synthesis of a highly stable cationic covalent organic framework for efficient and selective removal of $ReO_4^-/^{99}TcO_4^-$

Changxia Li,*[a,b] Justyna Florek,[b] Patrick Guggenberger [b,c] and Freddy Kleitz*[b]

[a.] School of Chemistry and Molecular Engineering, Nanjing Tech University, 211816 Nanjing, China

[b.] Department of Functional Materials and Catalysis, Faculty of Chemistry, University of Vienna, 1090 Vienna, Austria

[c.] Vienna Doctoral School in Chemistry (DoSChem), University of Vienna, 1090 Vienna, Austria

*E-mail: changxia.li@univie.ac.at; freddy.kleitz@univie.ac.at

1. Experimental methods

1.1. Characterization methods

Wide-angle powder X-ray diffraction (XRD) patterns were carried out on a PANalytical EMPYREAN powder diffractometer equipped with the PIXcel^{3D} detector (Malvern PANalytical, United Kingdom) in reflection geometry (Bragg–Brentano HD) using Cu K$\alpha_{1/2}$ radiation, operated at a voltage of 45 kV and a tube current of 40 mA, with a fixed divergence slit of 0.05 mm. The measurements were performed in the continuous mode with a step size 2θ of 0.013° and a data time per step of 300 s. Nitrogen physisorption isotherms were measured at −196 °C on an Anton Paar Quantatech Inc. iQ2 instrument (Boynton Beach, FL, USA). The sample was degassed under vacuum at 150 °C for 15 h before the measurement. The specific surface area (S_{BET}) was calculated by using Brunauer-Emmett-Teller (BET) method on the adsorption isotherm. The pore size distribution plot was obtained from the adsorption branch of isotherms by the quenched solid density functional theory (QSDFT) model (slit/cylindrical pores, N_2 at 77 K on carbon). The CO_2 sorption measurement was performed at 25 °C on a Micromeritics 3FLEX (USA) sorption analyser. The sample was degassed under vacuum at 120 °C for 12 h before the measurement. Scanning electron microscopy (SEM) analyses were performed on a Zeiss Supra 55 VP electron microscope equipped with an EDS detector. The accelerating voltages are 5 kV and 20 kV for images and EDS mappings, respectively. Fourier transform infrared (FT-IR) spectra were measured on a Bruker VERTEX 70v FT-IR Spectrometer equipped with Specac Golden Gate ATR accessory. Thermogravimetric analysis (TGA) was recorded on a Netzsch instrument (STA 449-F3 Jupiter) from 25 to 800 °C at a heating rate of 10 °C min^{-1} in air atmosphere (20 mL min^{-1}). Solid-state magic angle spinning nuclear magnetic resonance spectroscopy (MAS NMR) spectra were performed on a Bruker Avance NEO 500 wide bore system (Bruker BioSpin, Rheinstetten, Germany). A 4 mm triple resonance magic angle spinning (MAS) probe was used. Cross-polarization (CP) was employed using a ramped contact pulse, thereby sweeping the proton radio frequency field from 50 to 100%. The resonance frequency for ^{13}C NMR was 125.78 MHz, the MAS rotor spinning was set to 14 kHz, and the CP contact time to 3 ms. The ReO_4^- concentrations in the solution were determined on an Agilent 7800 inductively coupled plasma mass spectrometer (ICP-MS).

1.2. Materials synthesis

1.2.1. Synthesis of Tp

108 mmol hexamethylenetetramine (15.1 g), 49 mmol phloroglucinol (6.0 g), and 90 mL trifluoroacetic acid were refluxed at 100 °C for 2.5 h under N_2. 150 mL HCl (3 M) was added slowly and the solution was heated at 100 °C for another 1 h. After cooling down, the solution was filtered through Celite and extracted with 350 mL of dichloromethane. After that, the solution was evaporated under reduced pressure to afford ~2.4 g of an off-white powder. Purification was carried out by sublimation.

1.2.2. Synthesis of the COF

PTSA (2.5g) and 856 mg DB were ground well, then 5 mL of water was added drop by drop. The mixture was ground thoroughly, then transferred into a bottle and 15 mL of water was added. The mixture was shaken well in a vortex shaker for 5 min. Then, 315 mg of Tp was added and the mixture was shaken for another 20 min. The red mixture was transferred into the autoclave with another 5 mL of water and kept in the oven at 125 °C for 24 h. Then, the red precipitate was filtered and sequentially washed with 3M HNO_3 and water. Finally, the collected solid was Soxhlet extracted with THF and dried at 100 ^{o}C to get TpDB-COF in ~96% yield.

2. Adsorption experiments

2.1. Batch experiments

All the experiments were conducted at room temperature (~25 ^{o}C) using the batch sorption method. The removal percentage (%) and the sorption capacity at equilibrium q_e (mg g^{-1}) were calculated by the following equations:

$$Removal\ (\%) = \frac{C_0 - C_e}{C_0} \times 100\%$$

$$q_e = \frac{C_0 - C_e}{m} \times V$$

in which C_0 (mg g^{-1}) and C_e (mg g^{-1}) are the initial and equilibrium concentrations of adsorbate, V (mL) is the volume of solution, and m (g) is the mass of sorbent.

2.2. Sorption kinetics

10 mg of TpDB-COF was added to 10 ml of aqueous solution containing 28 ppm of ReO_4^-. The mixture was shaken for 30 s, 1 min, 3 min, 5 min, and 10 min, respectively. The suspension was separated using a 0.22 μm nylon membrane filter. The concentrations of metals remaining in the filtered solution were then analyzed by inductively coupled plasma mass spectrometry (ICP-MS).

2.3. Sorption isotherms

The ReO_4^- sorption isotherm experiments for TpDB-COF were conducted by varying the initial concentrations of ReO_4^- ranging from 28 to 2500 ppm with the solid/liquid ratio of 1 g L^{-1}. After being shaken for 14 h, the suspension was separated with a 0.22 μm nylon membrane filter and the concentrations of remaining ReO_4^- were determined by ICP-MS.

The adsorption isotherms were analyzed by applying the Langmuir isotherm model. The Langmuir isotherm model was expressed according to the following equation:

$$\frac{C_e}{q_e} = \frac{1}{K_L q_m} + \frac{C_e}{q_m}$$

where q_m is the maximum adsorption capacity of adsorbent (mg g^{-1}) and K_L is the Langmuir constant (L mg^{-1}). By fitting each isotherm with this model, values of q_m and K_L were obtained.

2.4. pH effect study

The solution pH was tuned by adding negligible volumes of diluted HNO_3 or NaOH. The effect of pH for the ReO_4^- uptake was carried out by ranging pH values from 1 to 13. 10 mg of TpDB-COF was added to 10 ml of an aqueous solution containing 200 ppm of ReO_4^-. After being shaken for 14 h, the resulting mixture was separated with a 0.22 μm nylon membrane filter and the concentrations of remaining ReO_4^- were determined by ICP-MS.

2.5. Anion competition study

The effect of competing ions was studied and carried out at the solid/liquid ratio of 1 g L^{-1}, room temperature. The effect of $H_2PO_4^-$, SO_4^{2-}, ClO_4^-, CO_3^{2-}, NO_3^-, or Cl^- was studied by loading 0.5 mM

NaH_2PO_4, Na_2SO_4, $NaClO_4$, Na_2CO_3, $NaNO_3$, or NaCl solution into a 0.5 mM ReO_4^- solution, respectively. The influence of excessive NO_3^- was screened by adding different concentrations of $NaNO_3$ (0.15 mM, 0.75 mM, 1.5 mM, 3 mM, and 15 mM) into a 0.15 mM ReO_4^- solution. The influence of excessive SO_4^{2-} was investigated by adding different concentrations of Na_2SO_4 (0.08 mM, 0.8 mM, 8.0 mM, 80 mM, and 480 mM) into a 0.08 mM ReO_4^- solution. After being shaken for 14 h, the suspensions were separated with a 0.22 μm nylon membrane filter and the concentrations of the remaining ReO_4^- were determined by ICP-MS.

2.6. Batch experiments in 1M NaOH and 1M HNO_3 system

2 mg, 4 mg, 10 mg, 20 mg or 40 mg of TpDB-COF were added into 2 ml of 1M NaOH or 1M HNO_3 solution containing 200 ppm of ReO_4^-, respectively. After shaking it for 14 h, the suspension was separated with a 0.22 μm nylon membrane filter and the concentrations of remaining ReO_4^- were determined by ICP-MS.

2.7. Recyclability study

The recyclability was measured in a neutral aqueous solution with an initial ReO_4^- concentration of 28 ppm and a 1 M HNO_3 aqueous solution containing 200 ppm ReO_4^-, respectively. In the first cycle, 60 mg TpDB-COF was immersed into the ReO_4^- aqueous solution and shaken for 14 h. The concentrations of the remaining ReO_4^- were determined by ICP-MS. After that, the adsorbed material was eluted using 60 ml of 1 M NaBr solution at room temperature overnight, followed by washing with distilled water three times. The regenerated material was used for the next cycling test.

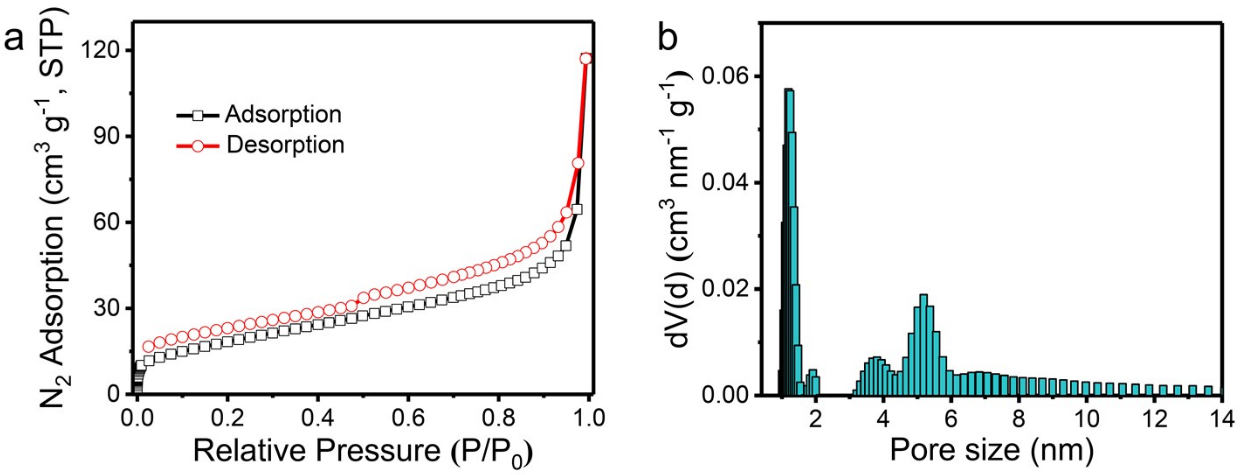

Figure S1. (a) N_2 adsorption-desorption isotherm (-196 °C) and (b) QSDFT pore size distribution of TpDB-COF.

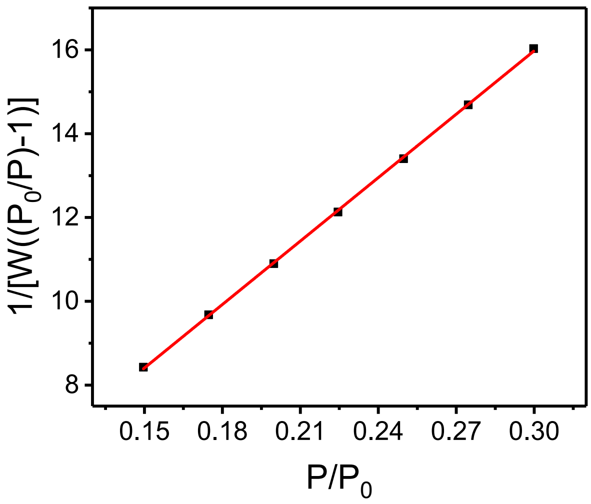

Figure S2. Linear range of the BET specific surface area plot calculated for the TpDB-COF N_2 physisorption isotherm.

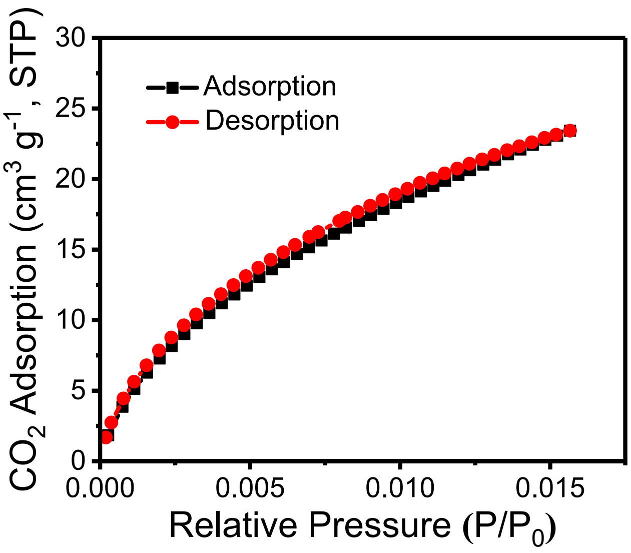

Figure S3. CO_2 adsorption-desorption isotherm of TpDB-COF measured at 25 °C.

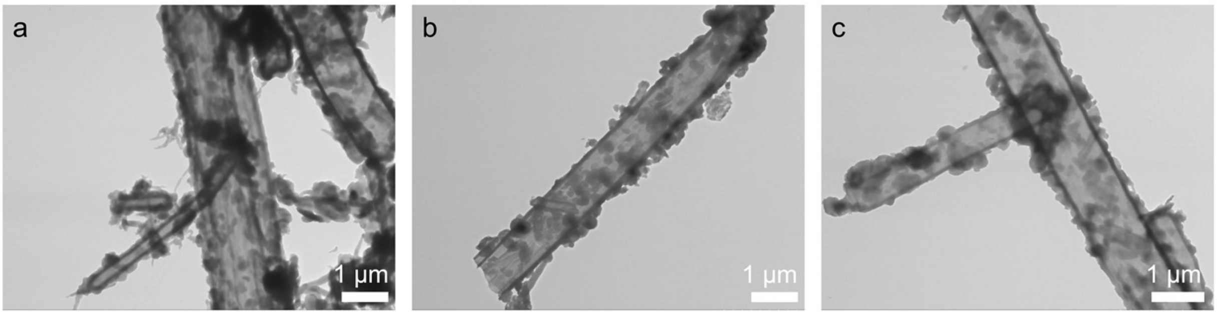

Figure S4. TEM images of (a) TpDB-COF, and TpDB-COF after treatment with (b) 3 M HNO_3 for 14 days and (c) 3 M NaOH for 4 days.

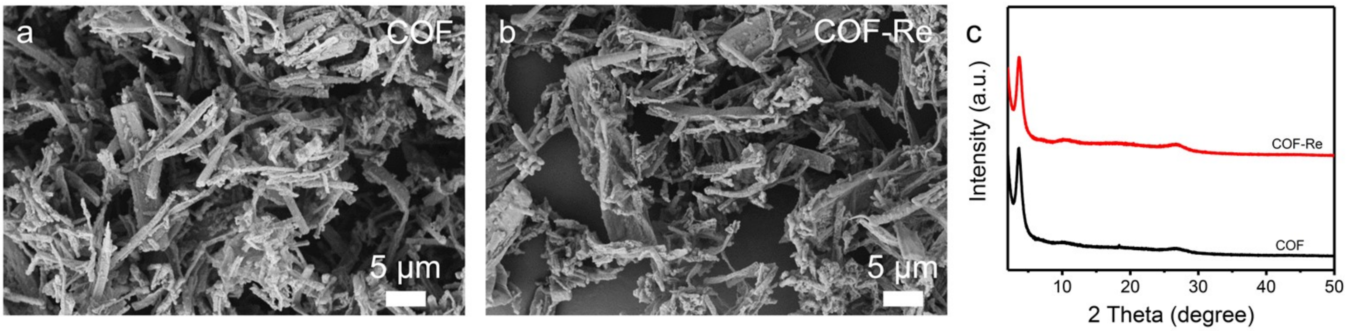

Figure S5. SEM images of (a) TpDB-COF, (b) TpDB-COF-Re, and (c) powder XRD patterns of COF and COF-Re.

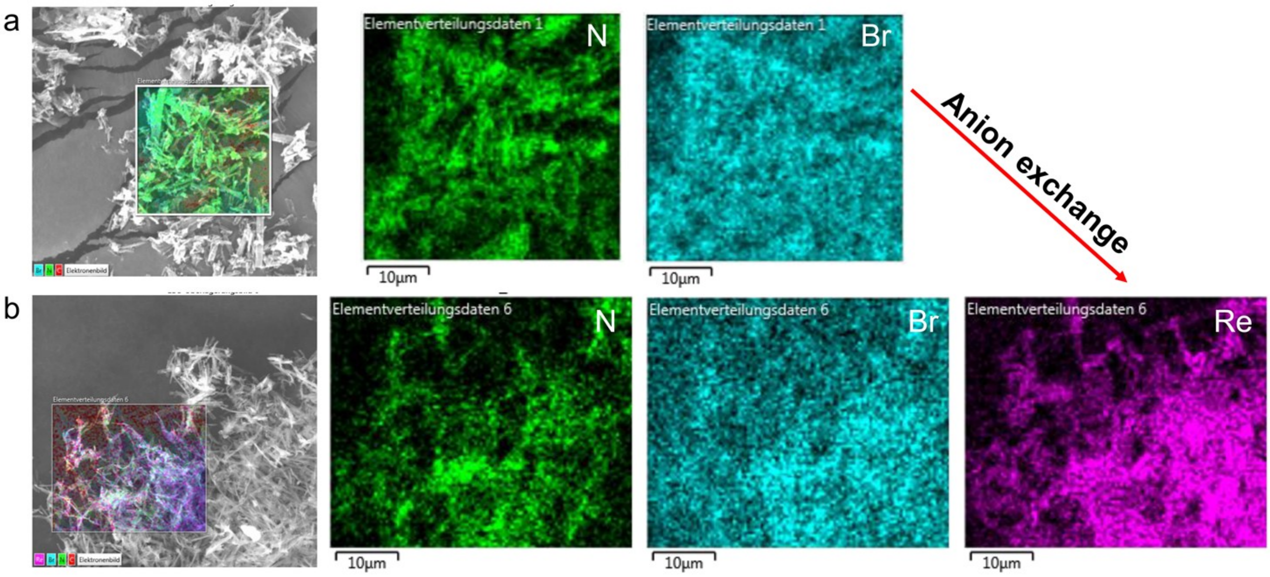

Figure S6. SEM and EDS mapping images of (a) TpDB-COF and (b) TpDB-COF-Re.

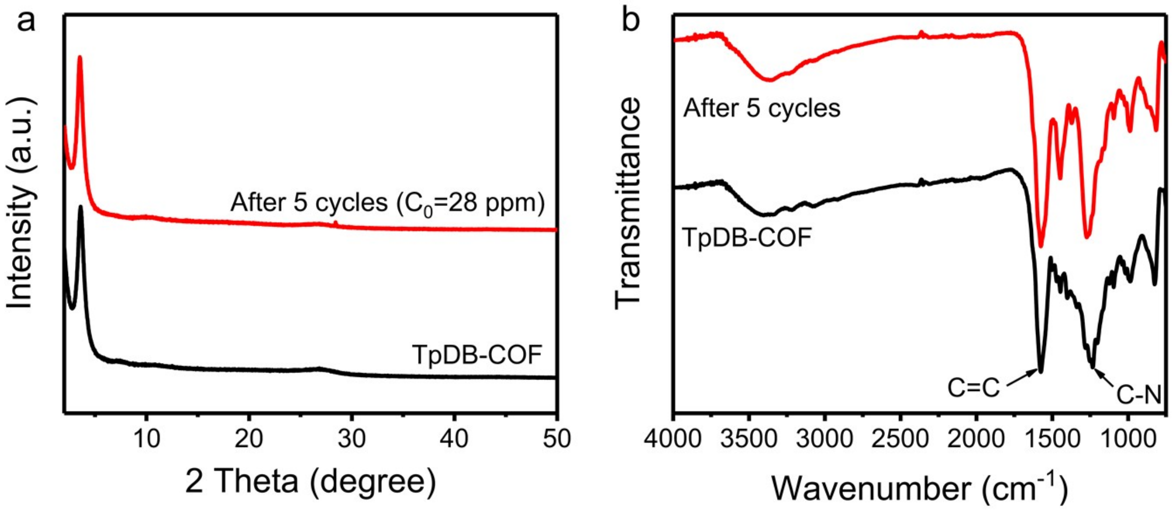

Figure S7. (a) Powder XRD patterns and (b) FT-IR spectra of fresh TpDB-COF and the sample after 5 cycles with initial concentration of ReO_4^- ~28 ppm (solid/liquid ratio of 1 mg mL^{-1}).

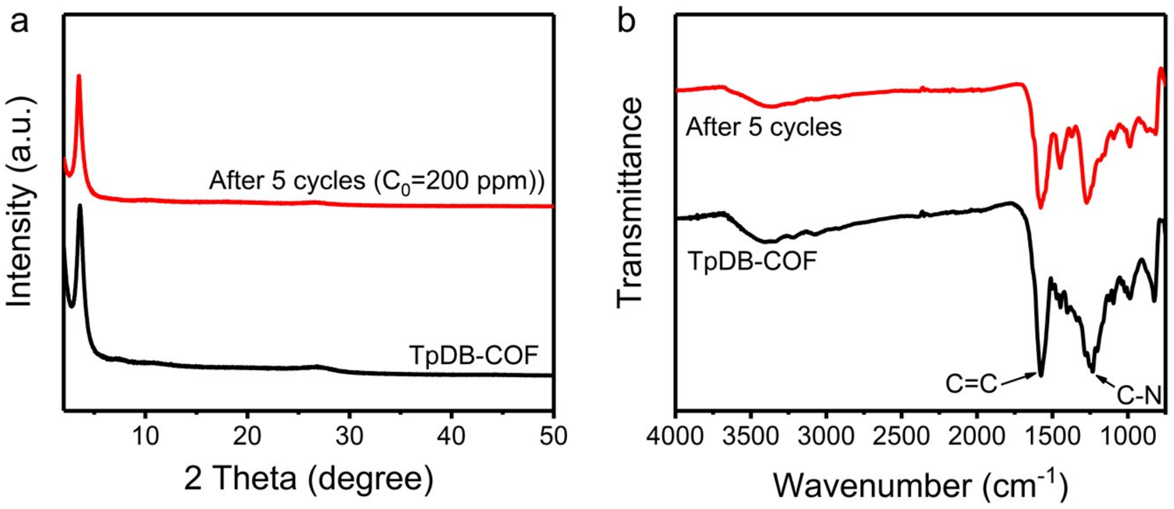

Figure S8. (a) Powder XRD patterns and (b) FT-IR spectra of fresh TpDB-COF and the sample after 5 cycles in 1 M HNO_3 with initial concentration of ReO_4^- ~200 ppm (solid/liquid ratio of 20 mg mL^{-1}).

Table S1. Comparison of reaction solvents, reaction time, product per batch, equilibrium time and adsorption capacities of TpDB-COF with other MOF/COF-based adsorbents.

Materials	Reaction solvents	Reaction time	Product per batch (g)	Adsorption equilibration time (min)	Adsorption capacity (mg g^{-1})	Refs
SCU-COF-1	1,2-dichlorobenzene	120 ^oC 6d	<0.025[a]	1	367.6	1
DhaTGCl	THF/H_2O	120 ^oC 3d	0.072	5	437	2
[C2vimBr]x %-TbDa-COF	1,2-dichlorobenzene/ n-butylalcohol	120 ^oC 3d	0.099	30	952	3
MOF-SCU-101	DMF, Ethanol, H_2O	100 ^oC 3d	0.491	10	247	4
UiO-66-NH2	DMF	120 ^oC 1d	0.65	~720	159	5
Poly(ionic liquids)@MOF	DMF	200 ^oC 1d	-	0.5	362	6
MOF-SCU-103	DMF/H_2O	140 ^oC 3d	0.065	5	318	7
Olefin-COF	Mesitylene/1,4-dioxane/ acetonitrile	150 ^oC 3d	0.032	1	726	8
Vinylene-3D COF	Mesitylene/1,4-dioxane	150 ^oC 3d	0.025	0.5	542.3	9
PS-COF-1	mesitylene/ ethanol	80 ^oC 3d	<0.08[a]	180	1262	10
PIL@MOF@ COF	n-butyl alcohol/o-dichlorobenzene	120 ^oC 3d	<0.245[a]	2	382	11
SCU-CPN-1	DMF	100 °C 4h (150 psi, 150W)	0.154	0.5	876	12
TpDB-COF	H_2O	125 ^oC 1d	1.12	1	570.4	This work

[a] The yield was not given. The calculation is based on the amount of reaction precursors.

Reference

1. L. He, S. Liu, L. Chen, X. Dai, J. Li, M. Zhang, F. Ma, C. Zhang, Z. Yang, R. Zhou, Z. Chai, S. Wang, *Chem. Sci.*, 2019, **10**, 4293.
2. H. J. Da, C. X. Yang and X. P. Yan, *Environ. Sci. Technol.*, 2019, **53**, 5212.
3. Y. Wang, M. Xie, J. Lan, L. Yuan, J. Yu, J. Li, J. Peng, Z. Chai and W. Shi, *Chem*, 2020, **6**, 2796.
4. L. Zhu, D. Sheng, C. Xu, X. Dai, M. A. Silver, J. Li, P. Li, Y. Wang, Y. Wang, L. Chen, C. Xiao, J. Chen, R. Zhou, C. Zhang, O. M. Farha and Z. Chai, T. E. Albrecht-Schmitt and S. Wang, *J. Am. Chem. Soc.*, 2017, **139**, 14873.
5. D. Banerjee, W. Xu, Z. Nie, L. E. Johnson, C. Coghlan, M. L. Sushko, D. Kim, M. J. Schweiger, A. A. Kruger, C. J. Doonan, P. K. Thallapally, *Inorg. Chem.*, 2016, **55**, 8241.
6. C.-P. Li, H.-R. Li, J.-Y. Ai, J. Chen and M. Du, *ACS Cent. Sci.*, 2020, **6**, 2354.
7. N. Shen, Z. Yang, S. Liu, X. Dai, C. Xiao, K. Taylor-Pashow, D. Li, C. Yang, J. Li, Y. Zhang, M. Zhang, R. Zhou, Z. Cai and S. Wang, *Nat. Commun.*, 2020, **11**, 5571.
8. W.-R. Cui, W. Xu, Y.-R. Chen, K. Liu, W.-B. Qiu, Y. Li and J.-D. Qiu, *J. Hazard. Mater.,* 2023, **446**, 130603.
9. C. R. Zhang, W. R. Cui, S. M. Yi, C. P. Niu, R. P. Liang, J. X. Qi, X. J. Chen, W. Jiang, X. Liu, Q. X. Luo and J. D. Qiu, *Nat. Commun.*, 2022, **13**, 7621.
10. M. Hao, Z. Chen, H. Yang, G. I. Waterhouse, S. Ma and X. Wang, *Sci. Bull.*, 2022, **67**, 924.
11. Z. Di, Z.-F. Liu, H.-R. Li, Z. Liu and C.-P. Li, *Inorg. Chem. Front.*, 2023, **10**, 952.
12. J. Li, X. Dai, L. Zhu, C. Xu, D. Zhang, M. A. Silver, P. Li, L. Chen, Y. Li, D. Zuo, H. Zhang, C. Xiao, J. Chen, J. Diwu, O. K. Farha, T. E. Albrecht-Schmitt, Z. Chai and S. Wang, *Nat. Commun.*, 2018, **9**, 3007.
